# E-cigarettes and smoking cessation: a prospective study of a national sample of pregnant smokers

**DOI:** 10.1186/s12889-019-7299-7

**Published:** 2019-07-18

**Authors:** Shawn C. Chiang, Lorien C. Abroms, Sean D. Cleary, Ichhya Pant, Lindsay Doherty, Nandita Krishnan

**Affiliations:** 1 Department of Prevention and Community Health, Milken Institute School of Public Health, The George Washington University, 950 New Hampshire Ave. NW, Washington, DC 20052 USA; 2 Department of Epidemiology, Milken Institute School of Public Health, The George Washington University, Washington, DC USA

**Keywords:** e-cigarettes, mHealth, pregnancy, smoking cessation, text messaging

## Abstract

**Background:**

Smoking during pregnancy has adverse health consequences for the mother and fetus. E-cigarettes could aid with smoking cessation but there is limited research on the prevalence and patterns of e-cigarette use, and their association with smoking cessation among pregnant smokers.

**Methods:**

We conducted a secondary analysis of a randomized controlled trial of a text-messaging program for smoking cessation among a U.S. national cohort of pregnant smokers (*n* = 428). Outcomes assessed were trajectories of e-cigarettes use from baseline to one-month follow-up, and longitudinal association between e-cigarette use at baseline and smoking cessation at one-month follow-up.

**Results:**

At baseline, 74 (17.29%) pregnant smokers used e-cigarettes in the past 30 days and 36 (8.41%) used e-cigarettes in the past 7 days. The primary reason stated for using e-cigarettes during pregnancy was for quitting. E-cigarette use between baseline and 1-month was inconsistent. Of 36 dual-users at baseline, 20 (55.56%) stopped using e-cigarettes by the 1-month follow-up and 14 initiated e-cigarette use. There was no evidence of an association between e-cigarette use at baseline and the primary smoking cessation outcome, 7-day point prevalence abstinence [adjusted odds ratio = 0.79, 95% confidence intervals = 0.33–1.92].

**Conclusions:**

A secondary analysis of a national sample of pregnant smokers indicates that use of e-cigarettes is inconsistent and is not associated with improved smoking cessation outcomes. There is an urgent need to further examine the risk and benefits of e-cigarette use, especially during pregnancy.

**Electronic supplementary material:**

The online version of this article (10.1186/s12889-019-7299-7) contains supplementary material, which is available to authorized users.

## Background

Electronic cigarettes (e-cigarettes), battery-operated devices that vaporize nicotine and produce an inhaled aerosol, have burgeoned into a billion-dollar industry in the United States (U.S.) with over $10 billion (US dollars) in revenues by 2017 [1]. Contributing to the increase in revenue has been growing awareness and use of e-cigarettes by the general population [1, 2]. As e-cigarette use continues to grow, more research on the health effects and patterns of e-cigarette use is needed to inform policies. Although e-cigarettes are marketed as being less harmful to health than regular cigarettes [3], and have shown potential to satisfy nicotine addiction while delivering fewer toxicants [4], controversy exists about the promotion, safety, and use of e-cigarettes [5, 6]. Some previous longitudinal studies indicate that there is a benefit for reduction or cessation of tobacco cigarettes [7–12], while others point to no or little benefit [13–16]. Studies have also shown that e-cigarettes could deliver potentially greater amounts of nicotine than tobacco cigarettes [15–18] and lead to renormalization of smoking [19].

Due to the high urgency to quit cigarette smoking during pregnancy and the potential efficacy of e-cigarettes as a smoking cessation device [7–12], pregnant women may be particularly interested in using e-cigarettes to assist with quitting or reducing cigarette smoking. Smoking during pregnancy has been shown to cause pregnancy complications and adverse fetal outcomes, such as preterm-related deaths, sudden infant deaths, and low birth-weights [20]. It is estimated that 8.4–10.2% of pregnant women smoke during their pregnancy in the U.S. [21]. Rates are also higher among women who have less than a high school education, Medicaid insurance, are of White or Native American ancestry, and are between the ages of 20–24 years old [21, 22]. Given the lack of conclusive evidence regarding the efficacy of e-cigarettes for smoking cessation and concerns about their harms, a better understanding of e-cigarette use by pregnant smokers is necessary.

There is a dearth of research conducted to date focusing on the effects of e-cigarette use among pregnant women and few estimates exist on the prevalence of e-cigarette use among pregnant women. A recent systematic review found that the prevalence of e-cigarette use during pregnancy ranges from 0.6 to 15% [23]. A 2017 study found that 8.54% (38/445) of pregnant women were dual users of tobacco cigarettes and e-cigarettes. The study also found that pregnant women view e-cigarettes as being safer than tobacco cigarettes [24], even though e-cigarettes are not approved as a smoking cessation or reduction aid by the U.S. Food and Drug Administration [24, 25].

Given the potential link between nicotine exposure and adverse fetal and pregnancy outcomes [26, 27], understanding the motivations for and patterns of e-cigarette use among pregnant women is critical. The objectives of this study were to (i) identify trajectories of e-cigarette use, and (ii) examine the longitudinal association between e-cigarette use and smoking cessation among a U.S. national cohort of pregnant smokers.

## Methods

### Study population and design

The sample for this analysis comes from the Quit4baby trial, a randomized controlled trial of text messaging for smoking cessation in pregnant women in the U.S. Recruitment for the study occurred between August, 2015 and February, 2016 [28, 29]. Participants (*N* = 508) were recruited from enrollees in Text4baby, the largest text messaging service for pregnant women and mothers in the United States. As such, all participants were receiving Text4baby text messages at the time of enrollment. Some participants were randomized to also receive Quit4baby [29]. A detailed recruitment process is published elsewhere [28]. This study was approved by the George Washington University Institutional Review Board.

To be eligible, participants had to have a cell phone for their personal use, be willing to receive text messages on their phone, be 14 years old or older, be pregnant at the time of enrollment and report having smoked at least one puff of cigarette in the past 2 weeks at the time of assessment [29]. Per IRB protocol, participants were consented over the phone verbally, and for minors, parents were required to give consent and minors gave assent. The current analyses are limited to a sub-sample from this study who completed the 1-month follow-up survey and were still pregnant at the time of the follow-up (*n* = 428 participants including 4 minors; 13 were removed because they were no longer pregnant and 67 were removed because they did not complete the 1-month survey).

### Measures

The primary cigarette smoking outcome for these analyses was self-reported 7-day point prevalence abstinence (PPA) at 1-month follow-up using the question, “Have you smoked a cigarette, even a puff, in the last 7 days?” (Additional file 1). Participants missing 7-day PPA were coded as smokers (*n* = 1). Secondary smoking outcomes included self-reported attempt to quit for longer than 1 day and changes in average cigarettes smoked per day (CPD) between 1-month and baseline. Changes in CPD was calculated by subtracting CPD at 1 month from CPD at baseline.

The main exposure variable of interest was use of e-cigarettes reported at baseline. E-cigarette use was assessed at both baseline and 1-month follow-up by asking “Have you used an e-cigarette, even once, in the past 7 days?” (Additional file 1). To track changes in e-cigarette use of the sample between baseline and 1-month follow-up, the sample was categorized into four different groups: “Continued non-users” (i.e. no e-cigarette use at both baseline and 1-month), “Continued users” (i.e. use of e-cigarette at both baseline and 1-month), “Stopped users” (i.e. use of e-cigarette at baseline, but stopped at 1-month) and “New users” (no e-cigarette usage at baseline, but initiated use of e-cigarette at 1-month).

Several variables potentially associated with smoking cessation and e-cigarette use were measured at baseline. Demographic characteristics included age, gestational age (in number of weeks), body mass index (BMI), race/ethnicity, employment, education, health insurance type, marital status, income, geographic region and intervention allocation (i.e. either Quit4baby + Text4baby; or Text4baby). Smoking variables included CPD at baseline, the Fagerstrom Test for Cigarette Dependence (FTCD) [30, 31] and motivation to quit smoking (1 = not at all, 7 = extremely). Additionally, we assessed the reasons for using e-cigarettes at baseline by listing out commonly reported reasons [1, 16, 24] (e.g. safer for me than regular cigarettes, to help me quit, tastes good/does not smell).

### Statistical analyses

Analyses for this study were conducted using SAS 9.4. Fisher Exact and Mann-Whitney U tests were used to compare groups of interest – cigarette/e-cigarette dual-users and cigarette-only users, with respect to baseline demographic and smoking characteristics of the sample. To assess the association between the use of e-cigarettes at baseline and smoking cessation outcomes at 1-month, three logistic and linear regression models were constructed. Model 1 was an unadjusted bivariate logistic/linear regression. Model 2 was a multivariable logistic/linear regression that controlled for intervention allocation. Despite no differences in e-cigarette use observed between groups for intervention allocation, Model 2 was constructed because the intervention was shown to have positive smoking cessation outcomes [29]. Model 3 was a multivariable logistic/linear regression that controlled for baseline characteristics (i.e. race/ethnicity) that were found to be significantly different between groups in addition to intervention allocation.

## Results

### Sample characteristics

Table 1 presents the overall baseline sample characteristics as well as characteristics for both e-cigarette/cigarette dual users and cigarette-only users. The sample (*n* = 428) was on average 26.39 years old (Standard Deviation (SD) = 5.80), 18.07 weeks pregnant (SD = 7.75) and had an average BMI of 27.73 (SD = 7.77). They were primarily non-Hispanic White (63.00%), unemployed (67.53%), with Medicaid or Medicare insurance (80.52%), with a high school degree or less (60.28%) and from the south (55.61%). At baseline, the sample smoked an average of 7.50 cigarettes per day (SD = 6.32), had an average FTCD score of 2.85 (SD = 2.28), and had a motivation to quit score of 6.04 out of 7 (SD = 1.31). There were no significant differences in demographic and smoking characteristics between participants lost to follow up and participants who completed the 1-month follow-up.

**Table 1:** Baseline characteristics of sample

Characteristics	E-Cigarette & Cigarette dual user (*n* = 36)	Cigarette user (*n* = 392)	All (*n* = 428)
Age, M (SD)	26.86 (4.50)	26.35 (5.91)	26.39 (5.80)
Gestational age, in weeks, M(SD)	18.28 (7.48)	18.05 (7.79)	18.07 (7.75)
Body mass index (BMI), M(SD)	26.84 (5.45)	27.81 (7.95)	27.73 (7.77)
Race/Ethnicity^a^			
White	30 (83.33)	239 (61.13)	269 (63.00)
Black/African-American	4 (11.11)	100 (25.58)	104 (24.36)
Other	2 (5.56)	52 (13.30)	54 (12.65)
Education			
< 12 grade, no high school diploma	6 (16.67)	110 (28.06)	116 (27.10)
High school graduate, GED^b^ or equivalent	12 (33.33)	130 (33.16)	142 (33.18)
Some college	16 (44.44)	115 (29.34)	131 (30.61)
College degree	2 (5.56)	37 (9.44)	39 (9.11)
Employment status			
Working part/full-time	13 (36.11)	125 (32.13)	138 (32.47)
Not working	23 (63.89)	264 (67.87)	287 (67.53)
Annual household income			
< $15,000	22 (62.86)	209 (54.43)	231 (55.13)
$15,001–$30,000	8 (22.86)	121 (31.51)	129 (30.79)
> $30,000	5 (14.29)	54 (14.06)	59 (14.08)
Marital status			
Single, never married	12 (33.33)	156 (39.90)	168 (39.34)
Living with significant other	9 (25.00)	128 (32.74)	137 (32.08)
Married	8 (22.22)	80 (20.46)	88 (20.61)
Divorced/Separated/Widowed	7 (19.44)	27 (6.91)	34 (7.96)
Health insurance			
Medicaid/Medicare	31 (86.11)	312 (80.00)	343 (80.52)
Private, veterans or other	3 (8.33)	64 (16.41)	67 (15.73)
None	2 (5.56)	14 (3.59)	16 (3.76)
Cigarettes smoked per day, M(SD)	8.44 (7.27)	7.41 (6.23)	7.50 (6.32)
Fagerstrom test for cigarette dependence (0–10), M(SD)	3.36 (2.54)	2.80 (2.25)	2.85 (2.28)
Motivation to quit (1–7), M(SD)	5.64 (1.53)	6.08 (1.28)	6.04 (1.31)
Region			
West	1 (2.78)	27 (6.89)	28 (6.54)
Mid-west	6 (16.67)	94 (23.98)	100 (23.36)
Northeast	2 (5.56)	60 (15.31)	62 (14.49)
South	27 (75.00)	211 (53.83)	238 (55.61)
Allocated to intervention arm	20 (55.56)	188 (47.96)	208 (48.60)

^a^Significant difference (*p* ≤ 0.05) was observed between control and intervention group based on fisher exact test

^b^*GED* General Educational Development test

Note: Data are presentated as n(%) unless otherwise noted

At baseline, 8.41% of participants (*n* = 36) reported using e-cigarettes in the past 7 days and 17.29% (*n* = 74) reported using e-cigarettes in the past 30 days. At 1 month, 7.00% of participants (*n* = 30) reported using e-cigarette in the past 7 days and 11.92% (*n* = 51) reported using e-cigarettes in the past 30 days. No differences in demographic variables were observed between e-cigarette dual users (past 7-day) and cigarette-only users except race/ethnicity, where a greater percentage of dual users were non-Hispanic White (83.33% vs. 61.13%; *p* ≤ .05). There was no significant difference in the proportion of e-cigarette dual users between the 67 participants lost to follow up (12.9%) and participants who completed the 1-month follow-up (8.41%), *p* > 0.05.

When asked about reasons for using e-cigarettes at baseline, 80.56% (*n* = 29) of women reported that they were using e-cigarettes to “help me quit”, followed by “they are safer for me than regular cigarettes” (41.67% or 15/36), “e-cigarette tastes good and does not smell” (38.89% or 14/36) and “safer for my baby than regular cigarettes” (36.11% or 13/36). A small number also reported they were using e-cigarettes due to “cost” (13.89% or 5/36) or because “friends and family use them” (5.56% or 2/36).

### E-cigarette use trajectory

E-cigarette use between baseline and 1-month varied for some participants. A summary of e-cigarette use trajectory can be found in Table 2. At baseline, 36 (8.41%) women used e-cigarettes in the past 7 days. At 1-month, 16 of these 36 women (44.44%) continued using e-cigarettes (“Continued”) and 20 of these 36 women (55.56%) stopped using e-cigarettes (“Stopped”). Additionally, of 392 e-cigarette non-users at baseline, 14 (3.57%) started using e-cigarettes by 1-month (“New”). The majority of the sample (95.66% or 375/392) remained e-cigarette non-users at both time points (“Continued non-users”). Three women (0.70%) had missing e-cigarette use status at 1-month.

**Table 2:** Trajectories of e-cigarette use from baseline to 1-month follow-up

			1-month follow-up^a^	
			Used e-cigarettes	Did not use e-cigarettes
Baseline	Used e-cigarettes	36 (8.41%)	Continued 16 (44.44%)	Stopped 20 (55.56%)
	Did not use e-cigarettes	392 (91.59%)	New 14 (3.57%)	Continued non-users 375 (95.66%)
	Total	428	30 (7.01%)	395 (92.29%)

^a^3 observations missing at 1-month follow-up

Among those four trajectory groups, “continued non-users” had the highest quit rate, with 26.4% (99/375) reporting 7-day PPA abstinence at 1-month, followed by “stopped users” (25.00% or 5/20), “continued users” (12.50% or 2/16) and “new users” (7.14% or 1/14).

### Logistic and linear regression models

Table 3 presents unadjusted and adjusted odds ratios for the association between e-cigarette use at baseline and smoking outcomes at 1-month follow-up. In the unadjusted model (Model 1), the odds of 7-day PPA were lower among participants who used e-cigarettes compared to those who did not use e-cigarettes, though differences were not significant (odds ratio [OR] = 0.70, 95% confidence interval [CI] = 0.30,1.64, *ns*). Additionally, no differences were observed between use of e-cigarette and attempt to quit for more than 1 day (OR = 1.09, 95% CI = 0.52, 2.28, *ns*) and differences in CPD (OR = − 0.029, 95% CI = − 1.75, 1.69, *ns*). In the multivariable models (Models 2 and 3), the association between e-cigarette usage and smoking outcomes were not statistically significant. A sensitivity analysis that included the 67 participants lost to follow up (coded as smokers) yielded similar results for the association between e-cigarette use at baseline and 7-day PPA at 1-month follow-up.

**Table 3:** Unadjusted and Adjusted Odds Ratios for the Association Between E-Cigarette Use and Smoking Outcomes

Outcome variables	E-cigarette & cigarette dual user (*n* = 36)	Cigarette user (*n* = 392)	Model 1		Model 2		Model 3	
			OR (95% CI)	*p*-value	AOR (95% CI)	*p*-value	AOR (95% CI)	*p*-value
Not smoked in past 7 days^a^, *n*(%)	7 (19.44)	101 (25.77)	0.70 (0.30, 1.64)	0.41	0.64 (0.27, 1.53)	0.31	0.79 (0.33, 1.92)	0.61
Quit for more than 1 day, *n*(%)	25 (69.44)	265 (67.60)	1.09 (0.52, 2.28)	0.82	1.04 (0.49, 2.20)	0.92	1.20 (0.56, 2.55)	0.64
Difference in CPD, M(SD)	−3.39 (5.54)	−3.36 (4.97)	−0.029 (−1.75, 1.69)	0.97	0.04 (−1.67, 1.76)	0.96	0.18 (−1.55, 1.91)	0.84

Note: Model 1 unadjusted, Model 2 adjusted for intervention allocation, Model 3 adjusted for intervention allocation and race/ethnicity; *OR* odds ratio, *AOR* adjusted odds ratio, *CPD* cigarettes per day

^a^3 observations missing

## Discussion

The current study is among the first to examine the use of e-cigarettes among pregnant smokers prospectively. In this national sample of pregnant smokers recruited in 2015 and 2016, 8.41% of pregnant cigarette smokers were found to also be concurrent e-cigarette users. E-cigarette users were found to be similar to non-users with the exception of being more likely to be of white ethnicity. More than half of those who used e-cigarettes at baseline had stopped using by 1-month follow-up. After adjusting for intervention assignment and demographic factors, no association was observed between e-cigarette use and cigarette smoking related outcomes.

The most prevalent reasons for using e-cigarette in our sample were to “help me quit smoking”, followed by “e-cigarettes are safer for me compared to regular cigarettes.” This is similar to findings from previous studies that examined perceptions of e-cigarette use among pregnant women [1, 24]. Given these perceptions despite unknown health implications of e-cigarettes, targeted research is needed to understand the causal relationship between exposure to e-cigarettes and pregnancy outcomes (i.e. birthweight, preterm delivery).

Similar to other U.S. based studies [13, 14, 24], the majority of e-cigarette users in this study were non-Hispanic White (83%). In this study, the prevalence of e-cigarette dual use was 8.41%. Although our sample was limited to smokers enrolled in a smoking cessation text messaging program and as such, prevalence estimates may not be generalizable to all pregnant smokers, the prevalence of e-cigarette dual use in our sample mirrors estimates from a recent 2017 U.S.-based study, which found that 8.54% of pregnant women were e-cigarette dual users [24]. Additionally, in 2017, 2.8% of U.S. adults were current e-cigarette users and among U.S. adults aged 18–24, a group that’s most likely to be current e-cigarette users, the percentage was 5.2% [32]. Findings from our study suggest that rates of e-cigarette use among pregnant women may exceed those of general tobacco cigarette users in the U.S., and that e-cigarette use may be initiated during pregnancy.

Although e-cigarettes could benefit adult smokers if used as a complete substitute for combustible tobacco smoking [9–12], evidence of the effectiveness of e-cigarettes as a cessation aid is still inconclusive [33]. In this national sample of pregnant smokers, we found no evidence that e-cigarette use was associated with cigarette smoking related outcomes in the short term. However, recent randomized trials have shown that e-cigarettes are effective in aiding some adult smokers to quit or to reduce their cigarette consumption [9–12]. Further studies should consider recruiting a larger sample of pregnant women that use e-cigarettes and follow up for a longer period of time.

A strength of this study is the use of a large, national sample and therefore the findings can be generalized to pregnant women in the U.S. Our participants’ demographic characteristics – based on their predominantly White ethnicity, on public insurance (i.e. Medicaid), and having a household income of $30,000 or less per year- were found to match those of previous national samples of pregnant smokers in the U.S. [21].

Several limitations should be considered in this study. This study is a secondary analysis from an intervention study on quitting smoking. All participants were receiving health related text messages and therefore it is likely that the participants studied are more health-oriented than the general population of pregnant smokers. Additionally, as the number of e-cigarette users in our sample was relatively small, estimates may be unreliable. As with all self-reported measures, social desirability bias could be a limitation. Future studies may benefit from more targeted outreach of cigarette and e-cigarette dual users to examine the association between e-cigarette use and tobacco cigarette cessation. This study also did not collect information about the extent of e-cigarette use, frequency and duration of use, and the concentration of nicotine fluid in e-cigarettes. This information is critical to accurately classify participants as dual users as well as to have a comprehensive understanding of the safety and efficacy of e-cigarettes as a smoking cessation aid for this particular population. As a result, these findings should be interpreted with caution. Future research with appropriate measures is needed to further determine whether and how e-cigarettes can be an effective tobacco cigarette cessation or reduction aid for pregnant smokers.

## Conclusions

These data are among the first to provide estimates for the prevalence of e-cigarette use during pregnancy, their trajectory of use and their association with smoking cessation for pregnant smokers. This secondary analysis of a national trial of pregnant smokers provides some indication that use of e-cigarettes to quit smoking may be common in pregnant smokers but found no association with improved smoking cessation outcomes. There is an urgent need to further examine the risk and benefits of e-cigarette use, especially during pregnancy.

## Additional file

Additional file 1: Questionnaires. Baseline and 1-month follow-up questionnaires. (PDF 144 kb)

## Data Availability

The data included in the manuscript are not publicly available per ethical guidelines of the protocol, but are available via corresponding author given appropriate ethical approvals are obtained.

## Abbreviations

BMI: Body mass index
CI: Confidence interval
CPD: Cigarettes per day
FTCD: Fagerstrom Test for Cigarette Dependence
OR: Odds ratio
PPA: Point prevalence abstinence
SAS: Statistical Analysis System
SD: Standard deviation
U.S.: United States
