# Supplementary material for: E-cigarettes and smoking cessation: a prospective study of a national sample of pregnant smokers
Source: BMC Public Health. 2019 Jul 18;19:964. doi: 10.1186/s12889-019-7299-7 (PMC6637539; doi:10.1186/s12889-019-7299-7)
Supplement: Supplementary file 1 — Questionnaires. Baseline and 1-month follow-up questionnaires. (PDF 144 kb) [file 12889_2019_7299_MOESM1_ESM.pdf]

Quit4baby Baseline

Record ID ______________________________

Hi this is xx from George Washington University. I'm calling because you replied to text4baby about helping us test new messages. Are you still interested in learning more about our study?
- ◯ Yes
- ◯ No

If No, We're sorry you are no longer interested. You will continue to receive the text messages from text4baby. You can call 800-QUIT-NOW (800-784-8669) for free help at any time, even if you called before, and talk to a counselor for get free support and advice on how to quit. There are also many great resources for quitting smoking at http://www.smokefree.gov.

Ok, great! If you're eligible and interested, you will take a phone survey and get a giftcard for each interview completed. Do you have a few mins now to see if you are eligible?
- ◯ Yes
- ◯ No

Is there a better time to call?
- ◯ Yes
- ◯ No

Are you currently pregnant?
- ◯ Yes
- ◯ No INELIGIBLE (If no and it comes up that mom has suffered a pregnancy loss, here is a resource: First Candle is a 24-hour, toll-free hotline for parents who have experienced a pregnancy loss or the death of a baby. Telephone: (800) 221-7437.)

How old are you? ______________________________
((years))

Is participant 14 years or older?
- ◯ Yes
- ◯ No (INELIGIBLE)

Do you have a cell phone for your personal use?
- ◯ Yes
- ◯ No (INELIGIBLE)

The messages you receive from Text4Baby will always be free but we may send you some study related texts where standard rates would apply. Do you still want to participate?
- ◯ Yes
- ◯ No (INELIGIBLE)

Which of the following statements best describes you?
- ◯ I've never been a regular cigarette smoker- (INELIGIBLE)
- ◯ I used to be a regular cigarette smoker, but I wasn't smoking around the time I found out I was pregnant (INELIGIBLE)
- ◯ I have quit smoking cigarettes since finding out I was pregnant
- ◯ I smoke cigarettes every once in a while
- ◯ I smoke cigarettes regularly now, but less than before finding out I was pregnant
- ◯ I smoke cigarettes regularly now, about the same as before finding out I was pregnant

When did you last smoke a cigarette, even a puff? (not eligible if not within last 2 weeks) ______________________________
((M-D-Y))

Is participant eligible?
- ◯ Yes
- ◯ No

Thank you so much for you time. We're sorry we will not be able to include you in our study. You will continue to receive the text messaging program from Text4baby.

[If smoker but not eligible] There are many great resources for quitting smoking at http://www.smokefree.gov. For more help, you can also call a quitline at 1-877-44U-QUIT (1-877-448-7848) to talk to a counselor and get free advice on how to quit.

Before we move on to the first survey, I need to read you the consent for study participation.

Is participant 18 years of age or older? ◯ Yes ◯ No

INFORMED CONSENT

INTRODUCTION
You are invited to take part in a research study being conducted by Dr. Lorien Abroms at The George Washington University School of Public Health. Dr. Lorien Abroms works closely with Text4Baby. The study is funded by the National Institutes of Health. Dr. Abroms may make money from this program if it is successful.

You are being asked if you want to take part in this study because you said you are pregnant and a current smoker or recent quitter. Joining the study is completely up to you. If you decide to join, you can decide to stop at any time. You will continue to receive Text4Baby messages.

PURPOSE
The purpose of this study is to evaluate a new way to help moms quit smoking during pregnancy. This study is important because text messaging has not been studied in pregnant smokers.

PROCEDURES
If you agree to join, you will randomly be placed in one of two groups:
(1)The first group will continue to receive Text4baby messages.
(2)The second group will continue to receive Text4baby messages plus quit smoking text messages and may be offered a mobile phone application "app."

All of the text messages will come from 511411 and be free to you.

You will be asked to do up to 4 interviews by phone or by computer. The first interview will be today then again in 1 month and 3 months, and possibly 6. The interviews will each take less than 30 minutes of your time. You may also be asked to provide a saliva (spit) sample at the end of the study to see if you are still smoking.

The total amount of time you will spend in this study is approximately 2.5 hours over 6 months. Your time will be spent on interviews, plus the time to read and respond to text messages.

RISKS & CONFIDENTIALITY
You may feel some emotional stress/discomfort answering the interview questions. You are free to skip any questions or stop the interview at any time.

There is a small chance that someone not on our research team could find out that you took part in the study or somehow connect your name with the information we collect about you.

The following steps are being taken to reduce this risk: 1) all of the interview data will be kept private 2) we also password protect and securely store all study data.

The research team will look at your responses to Text4baby as well as any smoking related messages.

The records of this study will be kept private. In any published articles or presentations, we will not include any information that will make it possible to identify you as a subject. Your records for the study may be reviewed by the National Institutes of Health and by departments of the University, and partners responsible for overseeing research safety.

BENEFITS
Taking part in this research may or may not provide you or your baby direct benefit; however, the benefit to society will be a better understanding of the effectiveness of text messaging as a way of helping pregnant smokers quit.

COMPENSATION
You will receive 1 gift card after each completed interview. After the first interview you will receive $15, after the second $20, after the third interview $25, and after the fourth, if there is a 4th, another $25. This mean you could earn up to $85 in gift cards for taking part in this study and an additional $25 if we ask you to do a saliva test at the end of the study.

QUESTIONS
If you have questions, concerns, complaints, or think you have been harmed you can contact the Principal Investigator for this study, Dr. Lorien Abroms, at 202-994-3518, or for questions regarding your rights as a participant in human research call the GWU Office of Human Research at 202-994-2715.

DOCUMENTATION OF VERBAL CONSENT
After you agree to participate, the research team will provide you with a copy. Please keep it in case you want to read it again or call someone about the study.

INFORMED ASSENT

INTRODUCTION

You are invited to take part in a research study being conducted by Dr. Lorien Abroms at The George Washington University School of Public Health. Dr. Lorien Abroms works closely with Text4Baby. The study is funded by the National Institutes of Health. Dr. Abroms may make money from this program if it is successful.

You are being asked if you want to take part in this study because you said you are pregnant and a current smoker or recent quitter. Joining the study is completely up to you. If you decide to join, you can decide to stop at any time. You will continue to receive Text4Baby messages.

PURPOSE
The purpose of this study is to evaluate a new way to help moms quit smoking during pregnancy. This study is important because text messaging has not been studied in pregnant smokers.
PROCEDURES
If you agree to join, you will be placed in one two groups:
(1) The first group will continue to receive Text4baby messages.
(2) The second group will continue to receive Text4baby messages plus quit smoking text messages and may be offered a mobile phone application "app."
All of the text messages will come from 511411 and be free to you.

You will be asked to do up to 4 interviews by phone or by computer. The first interview will be today. Then we will contact you again in 1 month and 3 months, and possibly 6 months, for follow-up interviews. The interviews will each take less than 30 minutes of your time. You may also be asked to provide a saliva (spit) sample at the end of the study to see if you are still smoking.

The total amount of time you will spend in this study is about 2.5 hours over 6 months. Your time will be spent on interviews, plus the time to read and respond to text.

RISKS & CONFIDENTIALITY
You may feel uncomfortable answering the interview questions. You are free to skip any questions or stop the interview at any time.

There is a small chance that someone not on our research team could find out that you took part in the study or somehow connect your name with the information we collect about you.

The following steps are being taken to reduce this risk: 1) all of the interview data will be kept private; 2) we also password protect and securely store all study data.

The research team will look at your responses to Text4baby as well as any smoking related messages.

The records of this study will be kept private. In any articles or presentations, we will not include any information that will make it possible to identify who you are. Your records for the study may be reviewed by the National Institutes of Health and by departments of George Washington University, and people responsible for overseeing research safety.

BENEFITS
Taking part in this research may or may not provide you or your baby direct benefit; however, the benefit to society will be a better understanding of the effectiveness of text messaging as a way of helping pregnant smokers quit.

COMPENSATION
You will receive 1 gift card after each completed interview. After the first interview you will receive $15, after the second $20, after the third $25, and after the fourth if there is a fourth, another $25. This mean you could earn up to $85 in gift cards for taking part in this study and an additional $25 if we ask you to do a saliva test at the end of the study.

QUESTIONS
If you have questions, concerns, complaints, or think you have been harmed you can contact the Principal Investigator for this study, Dr. Lorien Abroms, at 202-994-3518. For questions regarding your rights as a participant in human research call the GWU Office of Human Research at 202-994-2715.

DOCUMENTATION OF VERBAL CONSENT
After you agree to participate, the research team will provide you with a copy. Please keep it in case you want to read it again or call someone about the study.

Do you agree to be part of this study? ◯ Yes ◯ No

Do you have any questions before we continue?	◯ Yes<br>◯ No
When is your birth date?	________________<br>((M-D-Y))
When is your due date?	________________<br>((M-D-Y))
What is the highest level of education you have received?	◯ 12th grade or less with no high school diploma<br>◯ GED or equivalent<br>◯ High School graduate<br>◯ Some College<br>◯ Associate's Degree<br>◯ Bachelor's Degree<br>◯ Master's, Doctoral degree or professional school degree
Do you currently work?	◯ Part Time<br>◯ Full Time<br>◯ Not at all
What type of health insurance do you have (READ CHOICES ALOUD)?	◯ None<br>◯ Medicaid/Medicare<br>◯ Veterans or Military<br>◯ Private insurance/from employer<br>◯ Other
Other:	________________
What is your marital status? (READ ALL CHOICES OUT LOUD)	◯ Single, never married<br>◯ Living with significant other<br>◯ Married<br>◯ Divorced/Separated<br>◯ Widowed
What is your race?	☐ White<br>☐ Asian<br>☐ Native Hawaiian/Pacific Islander<br>☐ Black/African American<br>☐ American Indian/Alaska Native<br>☐ Other<br>(More than one can be selected)
Are you Hispanic, Latina or Spanish origin?	◯ Yes<br>◯ No<br>◯ Don't know
Which of the following best describes the total yearly income for your household? (Please stop me when I get to the category that best describes you).	◯ Up to $15,000<br>◯ $15,001 - $30,000<br>◯ $30,001 - $47,099<br>◯ $47,100 or more
How many children do you have?	________________<br>((put 0 if none))
(ONLY ASK IF PREVIOUSLY HAD CHILDREN) How long, if at all, did you breastfeed your baby (last pregnancy)?	◯ Not at all<br>◯ Less than 6 months<br>◯ 6 months to 2 years<br>◯ 2 years or more
How many people live in your household (including you)?	________________<br>(Number)
Do you have unlimited talk minutes?	◯ Yes<br>◯ No<br>◯ Don't know

Do you have unlimited texts?
- ◯ Yes
- ◯ No
- ◯ Don't know

What kind of cell phone do you have?
- ◯ Smartphone (like an iphone or android)
- ◯ Basic

On average, how many texts do you send/receive on any given day?

____
(Number)

How do you pay your phone bill?
- ◯ Monthly
- ◯ Pay as you go
- ◯ Other

Have you lost cell phone service in the last year?
- ◯ Yes
- ◯ No
- ◯ Don't know

For how many days were you without service?

____
(Number)

How did you hear about Text4Baby?
- ☐ Friend/Family
- ☐ Doctor/nurse or other healthcare provider
- ☐ Health plan
- ☐ Health department
- ☐ TV/radio/magazine ad
- ☐ Facebook/Twitter
- ☐ Online
- ☐ Other

Other:

____

On a scale of 1 to 5, where 1 is not at all satisfied and 5 is extremely satisfied, how satisfied are you with the Text4Baby text messages you receive?
- ◯ 1. Not at all Satisfied
- ◯ 2
- ◯ 3
- ◯ 4
- ◯ 5 Extremely Satisfied

Have you smoked a cigarette, even a puff, in the last 30 days?
- ◯ Yes
- ◯ No

Have you smoked a cigarette, even a puff, in the last 7 days?
- ◯ Yes
- ◯ No

How often do you smoke?
- ◯ Every day or almost every day
- ◯ Most days
- ◯ Some days
- ◯ Rarely

How soon after you wake up do you (did you) smoke your first cigarette?
- ◯ Within 5 min
- ◯ Within 6-30 min
- ◯ Within 31-60 min
- ◯ After 60 min

Do you (did you) find it hard not to smoke in places where it is not allowed?
- ◯ Yes
- ◯ No

What cigarette is (was) the most difficult to give up?
- ◯ The first in the morning
- ◯ Any other

Do you (did you) smoke more frequently during the first hours after waking up than during the rest of the day?
- ◯ Yes
- ◯ No

Do you (did you) smoke when you are so ill that you are in bed for most of the day?
- ◯ Yes
- ◯ No

On average, how many cigarettes per day did you smoke before you discovered you were pregnant?	______________ (Number)
On average, how many cigarettes do you (did you) smoke per day now (or after you were pregnant but before you quit)?	______________ (Number)
Not counting yourself, how many other people in your household smoke?	______________
Does this include your spouse/partner smoking?	◯ Yes<br>◯ No
In the past 30 days have you been exposed to tobacco smoke in your workplace?	◯ Yes<br>◯ No
Do you currently smoke or allow others to smoke in your home?	◯ Yes<br>◯ No
Do you have a car?	◯ Yes<br>◯ No
Do you currently smoke or allow others to smoke in your car?	◯ Yes<br>◯ No
Since you found out you were pregnant, have you used medications to help you try to quit smoking (Nicotine gum, Zyban)?	◯ Yes<br>◯ No
Which ones?	______________
Which other tobacco products have you used in the past month besides cigarettes?	☐ Cigarettes (automatically check)<br>☐ Cigars<br>☐ Bidis<br>☐ e-cigarettes or e-vapes, which may look like regular cigarettes but are battery-powered and produce vapor instead of smoke like NJOY and Blu.<br>☐ Chewing Tobacco<br>☐ Dissolvable Tobacco<br>☐ Hookah<br>☐ Other<br>(Check all that apply)
Other	______________
How often do you use e-cigarettes?	◯ Everyday<br>◯ Some Days<br>◯ Rarely<br>◯ Never
Have you used an e-cigarette, even once, in the past 30 days (auto check is selected above)?	◯ Yes<br>◯ No
Have you used an e-cigarette, even once, in the last 7 days?	◯ Yes<br>◯ No
On days that you use e-cigarettes, how many puffs from the e-cigarette do you usually take?	______________
The next set of questions are about the reasons people use e-cigarettes. Please let us know which of the following are reasons that you use e-cigarettes:	☐ Cost<br>☐ Looks cool<br>☐ They are safer for me than regular cigarettes<br>☐ They are safer for my baby than regular cigarettes<br>☐ To help me quit<br>☐ Friends and family use them<br>☐ Tastes good/doesn't smell<br>☐ Other

Other: __________________________________

During the past 30 days, on or about how many days a week did you drink any alcoholic beverages? (DO NOT READ CHOICES OUT LOUD)

- ◯ One day per week
- ◯ Two days per week
- ◯ Three days per week
- ◯ Four days per week
- ◯ Five days per week
- ◯ Six days per week
- ◯ Seven days per week
- ◯ Less than once a week
- ◯ Not at all/not once

Consider a drink to be a can or bottle of beer, a 4-ounce glass of wine or one cocktail containing 1-ounce of liquor. On average, when you drink alcoholic beverages, how many drinks do you have each day?

____
((PLEASE SPECIFY A NUMBER. NO RANGES OR DECIMALS.))

Have you smoked marijuana in the past 30 days?

- ◯ Yes
- ◯ No

Have you smoked marijuana in the past 7 days?

- ◯ Yes
- ◯ No

In the last month, was there ever a period of time when you were feeling depressed or down or when you lost interest in pleasurable activities most of the day, nearly every day, for at least 1 week? (Note to researcher: If participant mentions suicidal ideation, notify PI and IRB immediately)

- ◯ Yes
- ◯ No

STRESS & COPING

The next set of statements is about how things have been going for you lately. On a scale of 1 to 5 where 1 is "never", 2 is "seldom", 3 is "occasionally", 4 is "often", and 5 is "most of the time", please tell me how often each of the following statements was true for you in the past month.

You felt overwhelmed.

- ◯ Never
- ◯ Seldom
- ◯ Occasionally
- ◯ Often
- ◯ Most of the time

You felt that you had more stress than usual.

- ◯ Never
- ◯ Seldom
- ◯ Occasionally
- ◯ Often
- ◯ Most of the time

You were pressured by others.

- ◯ Never
- ◯ Seldom
- ◯ Occasionally
- ◯ Often
- ◯ Most of the time

You were able to cope with unexpected problems.

- ◯ Never
- ◯ Seldom
- ◯ Occasionally
- ◯ Often
- ◯ Most of the time

You felt able to cope with stress.
- ◯ Never
- ◯ Seldom
- ◯ Occasionally
- ◯ Often
- ◯ Most of the time

Is there someone in your life you can turn to for support if you tried to quit smoking?
- ◯ Yes
- ◯ No

What is this person's relation to you?
- ☐ Spouse/Romantic Partner
- ☐ Friend
- ☐ Family Member
- ☐ None
- ☐ Other

WORRY & OUTCOME EXPECTATIONS

On a scale from 1-7 where 1 is "not at all worried" and 7 is "extremely worried", how worried are you that your health is being harmed by your current level of smoking?(For quitters) How worried were you before you quit that your health was being harmed by your current level of smoking?
- ◯ 1. Not at all worried
- ◯ 2
- ◯ 3
- ◯ 4
- ◯ 5
- ◯ 6
- ◯ 7. Extremely worried

On the same scale where 7 is "extremely worried", how worried are you that your baby's health is being harmed by your current level of smoking? (for quitters) How worried were you before you quit that your baby's health was being harmed by your current level of smoking?
- ◯ 1 Not at all worried
- ◯ 2
- ◯ 3
- ◯ 4
- ◯ 5
- ◯ 6
- ◯ 7 Extremely worried

On a scale from 1 to 7 where 1 is "very little" and 7 is "a great deal," how much do you think quitting will (has) improve(d) your own health during this pregnancy?
- ◯ 1 Very little
- ◯ 2
- ◯ 3
- ◯ 4
- ◯ 5
- ◯ 6
- ◯ 7 A great deal

On the same scale where 7 is "a great deal," how much do you think quitting will (has) improve(d) your baby's health during this pregnancy?
- ◯ 1 Very Little
- ◯ 2
- ◯ 3
- ◯ 4
- ◯ 5
- ◯ 6
- ◯ 7 A great deal

On a scale from 1 to 7 where 1 is not at all motivated and 7 is extremely motivated, how motivated are you to quit and stay quit after the baby is born?
- ◯ 1-not at all motivated
- ◯ 2
- ◯ 3
- ◯ 4
- ◯ 5
- ◯ 6
- ◯ 7-extremely motivated

On a scale from 1 to 7 where 1 is "not at all confident" and 7 is "extremely confident," how confident are you that you can quit smoking during this pregnancy?
- ◯ 1 Not at all confident
- ◯ 2
- ◯ 3
- ◯ 4
- ◯ 5
- ◯ 6
- ◯ 7 Extremely confident

Now I'm going to ask you some questions about your health.

Has your doctor or other medical professional told you that you have any of the following conditions? Say "yes" if you hear one that you have.

- ☐ Hypertension or Preeclampsia
- ☐ Diabetes
- ☐ Gestational Diabetes/Diabetes in Pregnancy
- ☐ Asthma
- ☐ Anemia
- ☐ Depression
- ☐ Anxiety
- ☐ Bipolar Disorder
- ☐ Preterm Delivery (baby born before 37 weeks)
- ☐ Fetal Growth Restriction
- ☐ Placental Abnormality (placenta previa or abruption)
- ☐ Other chronic disease or condition you regularly take medication for

(Check all that apply)

Other medical condition ______________________

Before this pregnancy, what was your weight in pounds? ______________________ (lbs)

What is your height? ______________________ (feet)

______________________ (inches)

Before you became pregnant this time, would you say....

- ◯ You wanted to have the baby
- ◯ You wanted to have a baby, but not at this time
- ◯ You had doubts about having a baby
- ◯ You did not want to have a baby

First Name ______________________

Last Name ______________________

Address ______________________

State: ______________________ (abbreviation)

Mobile Phone ______________________ (xxx-xxx-xxxx)

Other Phone ______________________ (xxx-xxx-xxxx)

What phone would you like to receive study related messages on?

- ◯ Mobile
- ◯ Other

Email ______________________ (Please spell that out for us)

Would you like your gift card sent through email or mail? (verify email/mailing address above)

- ◯ Email
- ◯ Mail

What type of gift card would you prefer?

- ◯ Babies 'R us
- ◯ Walmart
- ◯ Amazon

Please provide us with a name and phone number of a close friend, relative or roommate. We will only contact them if we are unable to get in touch with you or if you lose phone service in order to reach you for future surveys and get you the additional gift cards. __________________________________

Can we contact you for future studies?
- ◯ Yes
- ◯ No

(Internal) Is participant in the intervention or control group?
- ◯ Intervention
- ◯ Control

You are now being enrolled in Quit4Baby. The first step is picking a QuitDate. Picking a QuitDate is an important step in quitting smoking. It is best to pick a specific day in the next week. That way you will have enough time to prepare a quit plan. It is best to pick a QuitDate that is not too stressful a day, and a day when you tend to smoke a little bit less than other days. Mondays are usually good for quitting.When would you like to quit smoking?
- ◯ Soon
- ◯ Not Ready Yet
- ◯ I already Quit

Write date here: (QUITDATE MUST BE IN THE NEXT TWO WEEKS, IF NERVOUS ABOUT SETTING IT LET THEM KNOW THEY CAN ALWAYS RESET THEIR QUITDATE WITHIN THE PROGRAM). ___________________________

Enroll in Quit4baby: https://quit4baby.com/SignUp

You should receive a message from us within the next 5 minutes. If you don't receive a message, please give us a call back.

We are glad that you will be part of our study. We will be contacting you again in a month, for a follow up phone interview to see how you are doing. Please remember that we welcome your honest feedback. There are no wrong answers. In fact, the more honest you are with us, the higher the likelihood that your responses will help us understand pregnant smokers as a group.

If you have any questions please do not hesitate to call us at 202.994.3502. Thanks and talk to you soon!

Enrollment Date ___________________________

Who is completing the survey today?
- ◯ [redacted]
- ◯
- ◯
- ◯
- ◯
- ◯
- ◯
- ◯
- ◯
- ◯

Today's Date ___________________________

Recruitment Message Type
- ◯ Empathy
- ◯ Pride
- ◯ Free

Notes: __________________________________

Quit4baby 1 Month Survey

FOR INTERNAL USE: Is participant in control or intervention group?
- ◯ Control
- ◯ Intervention

Thanks again for participating in our study. This survey will take less than 30 minutes and I will send you a gift card once you complete.

Please answer the questions honestly, there are no wrong answers. All of the information you give will be kept private.

Are you still pregnant?
- ◯ Yes
- ◯ No

Did you have your baby?
- ◯ No
- ◯ Yes

FOR RESEARCHER ONLY: DON'T READ ALOUD (mark based on responses above)
- ◯ Still pregnant/had baby
- ◯ Lost baby

What is your baby's birthday?
____
((M-D-Y))

How much did your baby weigh at birth?(If twins, record both weights)
____
((8.5 for 8 lbs, 5 oz))

How many nights did you stay in the hospital when you had your baby?
____

After your baby was delivered, was he or she put in an intensive care unit (NICU)?
- ◯ Yes
- ◯ No
- ◯ Don't Know

After your baby was delivered, how long did he or she stay in the hospital?
- ◯ Less than 24 hours (less than 1 day)
- ◯ 24 to 48 hours (1 to 2 days)
- ◯ 3 to 5 days
- ◯ 6 to 14 days
- ◯ More than 14 days
- ◯ My baby was not born in a hospital
- ◯ My baby is still in the hospital

If it comes up mom has suffered a pregnancy loss here is a resource: First Candle is a 24-hour, toll-free hotline for parents who have experienced a pregnancy loss or the death of a baby. Telephone: (800) 221-7437.

Over the past 1-month have you lost cell phone service for text messages?
- ◯ Yes
- ◯ No

For how many days did you not have service?
____
(# of days)

Are you still receiving text messages from the Text4baby program, which sends general health and pregnancy tips?
- ◯ Yes
- ◯ No
- ◯ Don't know

Why did you stop receiving the messages? (If lost baby, ask if they cancelled their subscription and mark accordingly).
- ◯ I sent in STOP because I already quit
- ◯ I sent in STOP because I'm not pregnant anymore
- ◯ I cancelled my subscription for another reason
- ◯ I lost cell phone service
- ◯ I changed my phone number
- ◯ Don't know
- ◯ Other

Why did you send in STOP?
____

What is your new number?
____

When did you switch to this number? __________________________________

Other (please explain): __________________________________

At the time of study enrollment, did you begin to receive Quit4Baby messages, the program specific to helping people quit smoking?
- ◯ Yes
- ◯ No
- ◯ Don't know

Are you still receiving messages for the Quit4baby program?
- ◯ Yes
- ◯ No
- ◯ Don't know

Why did you stop receiving the messages? (If lost baby, ask if they cancelled their subscription and mark accordingly).
- ◯ I sent in STOP because I already quit
- ◯ I sent in STOP because I'm not pregnant anymore
- ◯ I cancelled my subscription for another reason
- ◯ I lost cell phone service
- ◯ I changed my phone number
- ◯ Don't know
- ◯ Other

Why did you send in STOP? __________________________________

What is your new number? __________________________________

When did you switch to this number? __________________________________

Other (please explain): __________________________________

What do you think about the number of text messages you received?
- ◯ Too many text messages
- ◯ Too few text messages
- ◯ Just right number of text messages

What proportion of the text messages did you read?
- ◯ All of them
- ◯ About 75% of them
- ◯ About 50% of them
- ◯ About 25% of them
- ◯ None of them

Which of the following have you used your phone for in the past week (Please say yes if you have used)?
- ☐ Facebook
- ☐ Instagram
- ☐ Twitter
- ☐ Health Apps
- ☐ Games
- ☐ Email
- ☐ Web
- ☐ Other

Other __________________________________

What health apps do you use?

____

Have you smoked a cigarette, even a puff, in the last 30 days?
- ◯ Yes
- ◯ No

Have you smoked a cigarette, even a puff in the last 7 days?
- ◯ Yes
- ◯ No

How often do you smoke?
- ◯ Every day or almost every day
- ◯ Most days
- ◯ Some days
- ◯ Rarely

On average, how many cigarettes do you smoke per day? __________________________________
((whole #))

The next question is about e-cigarettes or e-vapes, which may look like regular cigarettes but are battery-powered and produce vapor instead of smoke like NJOY and Blu.Have you used an e-cigarette in the last 30 days?

◯ Yes
◯ No

Have you used an e-cigarette in the past 7 days?

◯ Yes
◯ No

On the days that you use e-cigarettes, how many puffs do you typically take?

____

Do you currently smoke or allow others to smoke in your home?

◯ Yes
◯ No

Do you have a car?

◯ Yes
◯ No

Do you currently smoke or allow others to smoke in your car?

◯ Yes
◯ No

What was the longest continuous period (in days) that you did not smoke over the past 1 month?

____
(whole #'s only)

Since enrolling in this study, did you use any of the following to help you quit? (Check all that apply)

☐ A telephone help/quit line
☐ Talk to doctor or other health professional about quitting
☐ Support from partner, family or friend
☐ Self-help materials, books or videos
☐ Quit smoking website
☐ Quit smoking app
☐ Quit smoking text messages
☐ Electronic cigarettes
☐ Other

What is the name of the app?

____

What is the name of the text messaging program?

____

How many times did you talk to a quit counselor at the quit line?

____

Other (please explain):

____

Since enrolling the study, have you used medications to help you try to quit smoking (nicotine gum, Zyban)?

◯ Yes
◯ No

If yes, which medications have you used?

____

During the past 30 days, on or about how many days a week did you drink any alcoholic beverages? (DO NOT READ CHOICES ALOUD)

◯ One day per week
◯ Two days per week
◯ Three days per week
◯ Four days per week
◯ Five days per week
◯ Six days per week
◯ Seven days per week
◯ Less than once
◯ Never

Consider a drink to be a can or bottle of beer, a 4-ounce glass of wine, or one cocktail containing 1-ounce of liquor. On average, when you drink alcoholic beverages, how many drinks do you have each day? (PLEASE SPECIFY A NUMBER. NO RANGES OR DECIMALS.)

____

Have you smoked marijuana in the past 30 days?

◯ Yes
◯ No

Have you smoked marijuana in the past 7 days?
- ◯ Yes
- ◯ No

Now we would like to ask you questions about the Quit4Baby and Text4Baby program.

Now we would like to ask you questions about the Text4baby program.

Please rate how much you agree with the following statements where 1 is completely disagree and 5 is completely agree.

I would recommend the texts to a friend who was pregnant and smoking
- ◯ 1-Completely disagree
- ◯ 2-Disagree
- ◯ 3-Neither Agree nor Disagree
- ◯ 4-Agree
- ◯ 5-Completely agree

The texts gave me the confidence that I can quit smoking
- ◯ 1-Completely disagree
- ◯ 2-Disagree
- ◯ 3-Neither Agree nor Disagree
- ◯ 4-Agree
- ◯ 5-Completely agree

The texts felt like a trigger and made me want to smoke
- ◯ 1-Completely disagree
- ◯ 2-Disagree
- ◯ 3-Neither agree nor disagree
- ◯ 4-Agree
- ◯ 5-Completely agree

The texts made me feel like someone cared if I quit smoking.
- ◯ 1-Completely disagree
- ◯ 2-Disagree
- ◯ 3-Neither agree nor disagree
- ◯ 4-Agree
- ◯ 5-Completely agree

Is there anything else you did not like about the text messages?

____

Is there anything else you liked about the text messages?

____

Did you have any technical problems with any of the SMS features having glitches or not working properly? [If so, we will contact: support@voxiva.com with the issue and follow-up]

____

Was there anything confusing about receiving both the Text4Baby and Quit4Baby messages together?

____

(DO NOT READ: CLICK NO UNTIL APP IS AVAILABLE) Did you download the Quit4Baby App?
- ◯ Yes
- ◯ No

Over the past week, how many times did you visit the app?

____
(Whole # only)

Please rate how much you agree with the following statements where 1 is completely disagree and 5 is completely agree.

I would recommend the Quit4Baby app to a friend who was pregnant and smoking
- ◯ 1- Completely disagree
- ◯ 2-Disagree
- ◯ 3-Neither agree nor disagree
- ◯ 4- Agree
- ◯ 5- Completely Agree

Why did you rate it that way?

____

The Quit4Baby app gave me the confidence that I can quit smoking

- ◯ 1- Completely disagree
- ◯ 2-Disagree
- ◯ 3-Neither agree nor disagree
- ◯ 4- Agree
- ◯ 5- Completely Agree

Why did you rate it that way?

____

The Quit4Baby app made me feel like someone cared if I quit

- ◯ 1- Completely disagree
- ◯ 2-Disagree
- ◯ 3-Neither agree nor disagree
- ◯ 4- Agree
- ◯ 5- Completely Agree

Why did you rate it that way?

____

I liked the interactive tools (games, facts, trackers) provided by the Quit4Baby app

- ◯ 1- Completely disagree
- ◯ 2-Disagree
- ◯ 3-Neither agree nor disagree
- ◯ 4- Agree
- ◯ 5- Completely Agree

The Quit4baby app gave me good ideas on how to quit smoking

- ◯ 1- Completely disagree
- ◯ 2-Disagree
- ◯ 3-Neither agree nor disagree
- ◯ 4- Agree
- ◯ 5- Completely Agree

On a scale of 1 to 5, where 1 is very little and 5 is very much, how much did you like the craving tool in the app?

- ◯ 1-Very little
- ◯ 2
- ◯ 3
- ◯ 4
- ◯ 5- Very much

How many times have you used the craving tool in the past 2 weeks?

____
(whole # only)

On a scale of 1 to 5, where 1 is very little and 5 is very much, how much did you like the smoked tool in the app?

- ◯ 1-very little
- ◯ 2
- ◯ 3
- ◯ 4
- ◯ 5-very much

How many times have you used the smoked tool in the past 2 weeks?

____
(whole # only)

Is there anything else you liked or disliked about the Quit4Baby app?

____

Did you have any technical problems with any of the Quit4Baby app features having glitches or not working properly?

____

Do you have any other recommendations for how to make the Quit4Baby app better?

____

Now we want you to rate the helpfulness of the following Quit4baby messages on a scale of 1 to 5, where 1 is not at all helpful 5 is completely helpful. The messages are examples of ones you may have received from the Quit4baby program.

On a scale from 1-5 rate:1. Welcome Message: Welcome! You've taken a big step for you & your baby-congrats! Quit4baby will send tips & advice. Reply HELP for tech help; STOP to cancel. Let's start! Here are some tools for you: Text FACT to learn about the harms of smoking, text CRAVE for help with cravings, or GUIDE for more keywords.

- ◯ 1-Not at all helpful
- ◯ 2- Not helpful
- ◯ 3-Somewhat helpful
- ◯ 4-Helpful
- ◯ 5-Completely helpful

Why did you rate it that way?

____

How might this message be improved?

____

Ok, now let's pretend that you smoked so we sent you the following message:Quit4baby: Sorry to hear you smoked! Go back to being a non-smoker. Throw out all cigs (wet & put in trash) and stay smokefree. You can do it! Reply 1 to recommit and 2 if not ready to set quit date.How would you rate this message?

- ◯ 1- Not at all helpful
- ◯ 2- Not helpful
- ◯ 3- Somewhat helpful
- ◯ 4- Helpful
- ◯ 5- Completely helpful

Why did you rate it that way?

____

How might this message be improved?

____

Next, I'm going to ask you about your thoughts and feelings about smoking and quitting. On a scale from 1-7 where 1 is "not at all worried" and 7 is "extremely worried", how worried are you that your health is being harmed by your current level of smoking?

- ◯ 1-not at all worried
- ◯ 2
- ◯ 3
- ◯ 4
- ◯ 5
- ◯ 6
- ◯ 7-extremely worried

On a scale from 1-7 where 7 is "extremely worried", how worried are you that your baby's health is being harmed by your current level of smoking?

- ◯ 1-not at all worried
- ◯ 2
- ◯ 3
- ◯ 4
- ◯ 5
- ◯ 6
- ◯ 7-extremely worried

On a scale from 1 to 7 where 1 is "very little" and 7 is "a great deal," how much do you think quitting will (has) improved your own health during this pregnancy?

- ◯ 1-very little
- ◯ 2
- ◯ 3
- ◯ 4
- ◯ 5
- ◯ 6
- ◯ 7-a great deal

On a scale from 1 to 7 where 1 is "very little" and 7 is "a great deal," how much do you think quitting will (has) improved your baby's health during this pregnancy?

- ◯ 1-very little
- ◯ 2
- ◯ 3
- ◯ 4
- ◯ 5
- ◯ 6
- ◯ 7-a great deal

On a scale of 1 to 7, where 1 is not at all motivated and 7 is extremely motivated, how motivated are you to stay quit after the baby is born?

- ◯ 1: Not at all motivated
- ◯ 2
- ◯ 3
- ◯ 4
- ◯ 5
- ◯ 6
- ◯ 7: Extremely motivated

On a scale from 1 to 7 where 1 is "not at all confident" and 7 is "extremely confident," how confident are you that you can quit smoking?

- ◯ 1-not at all confident
- ◯ 2
- ◯ 3
- ◯ 4
- ◯ 5
- ◯ 6
- ◯ 7-extremely confident

Now let's confirm your contact information.First/last name: ____________________

Address: ____________________

Phone: ____________________

Alt phone: ____________________

E-mail address: ____________________

Should we use your email or mailing address to send you the gift card?

- ◯ Email
- ◯ Mailing

What type of gift card would you like?

- ◯ Walmart
- ◯ Babies 'R Us
- ◯ Amazon

1-Month Survey Date ____________________

Who is completing the survey today?

- ◯ [redacted]

Notes:

____

Thank you for be a part of the Baby & Me Study and for telling us about your experiences with the text messages. We will call you again in 2 months for another 30 min survey.

I will send you your gift card today and you should receive it within the next 2 weeks.

If you have any questions or concerns, please contact study staff at babyandme@gwu.edu or call 202-994-3502. Thanks and have a great day!
